# Panmixia defines the genetic diversity of a unique arthropod-dispersed fungus specific to *Protea* flowers

**DOI:** 10.1002/ece3.1149

**Published:** 2014-08-21

**Authors:** Janneke Aylward, Léanne L Dreyer, Emma T Steenkamp, Michael J Wingfield, Francois Roets

**Affiliations:** 1 Department of Botany and Zoology, Stellenbosch UniversityPrivate Bag X1, Matieland, 7602, South Africa; 2 Department of Science and Technology (DST)/National Research Foundation (NRF) Centre of Excellence in Tree Health Biotechnology (CTHB), University of PretoriaPretoria, 0002, South Africa; 3 Department of Microbiology and Plant Pathology, University of PretoriaPretoria, 0002, South Africa; 4 Department of Conservation Ecology and Entomology, Stellenbosch UniversityPrivate Bag X1, Matieland, 7602, South Africa

**Keywords:** Dispersal, *Knoxdaviesia*, ophiostomatoid, panmixia

## Abstract

*Knoxdaviesia proteae*, a fungus specific to the floral structures of the iconic Cape Floral Kingdom plant, *Protea repens*, is dispersed by mites phoretic on beetles that pollinate these flowers. Although the vectors of *K. proteae* have been identified, little is known regarding its patterns of distribution. Seed bearing infructescences of *P. repens* were sampled from current and previous flowering seasons, from which *K. proteae* individuals were isolated and cultured. The genotypes of *K. proteae* isolates were determined using 12 microsatellite markers specific to this species. Genetic diversity indices showed a high level of similarity between *K. proteae* isolates from the two different infructescence age classes. The heterozygosity of the population was high (0.74 ± 0.04), and exceptional genotypic diversity was encountered (Ĝ = 97.87%). Population differentiation was negligible, owing to the numerous migrants between the infructescence age classes (*N*_*m*_ = 47.83) and between *P. repens* trees (*N*_*m*_ = 2.96). Parsimony analysis revealed interconnected genotypes, indicative of recombination and homoplasies, and the index of linkage disequilibrium confirmed that outcrossing is prevalent in *K. proteae* (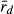 = 0.0067; *P* = 0.132). The high diversity and panmixia in this population is likely a result of regular gene flow and an outcrossing reproductive strategy. The lack of genetic cohesion between individuals from a single *P. repens* tree suggests that *K. proteae* dispersal does not primarily occur over short distances via mites as hypothesized, but rather that long-distance dispersal by beetles plays an important part in the biology of these intriguing fungi.

## Introduction

The ophiostomatoid fungi represent a unique and intriguing assemblage (Wingfield et al. 1993) that contain members specific to the floral parts within the infructescences (seed heads) of serotinous *Protea* L. species. These *Protea*-associated ophiostomatoid fungi are encountered primarily in the Core Cape Subregion (CCR; previously known as the Cape Floristic Region) of the Greater Cape Floristic Region (Wingfield et al. 1988; Wingfield and Van Wyk 1993; Marais and Wingfield 1994; Marais et al. 1998; Roets et al. 2005, 2006; Manning and Goldblatt 2012), but some have also been found where *Protea* species occur beyond this area (Marais and Wingfield 2001; Roets et al. 2008, 2010, 2013; Crous et al. 2012). Ophiostomatoid fungi are traditionally known as associates of bark beetles and mites that infest trees, and while many of these fungi are saprophytes, some are important plant pathogens (Six and Wingfield 2011; Seifert et al. 2013). The *Protea*-associated ophiostomatoid fungi do not appear to harm their plant or arthropod associates, and some have been shown to have a mutualistic relationship with their mycophagous mite vectors (Roets et al. 2007).

*Knoxdaviesia* M.J. Wingf., P.S. van Wyk & Marasas is an ophiostomatoid genus that includes three species occurring in *Protea* infructescences, namely *K. proteae* M.J. Wingf., P.S. van Wyk & Marasas, *K. capensis* M.J. Wingf. & P.S. van Wyk and *K. wingfieldii* (Roets & Dreyer) Z.W. de Beer & M.J. Wingf. The first two species are native to the CCR and have overlapping distributions (Marais and Wingfield 2001), while *K. wingfieldii* occurs in the KwaZulu-Natal Province (Crous et al. 2012). Whereas *K. capensis* is a generalist that has been found on various *Protea* hosts, *K. proteae* occurs exclusively in the infructescences of *P. repens* L. Despite its apparent lack of host specificity, *K. capensis* has never been encountered in *P. repens* (Wingfield and Van Wyk 1993; Marais et al. 1998; Roets et al. 2009b).

**Figure 1:**
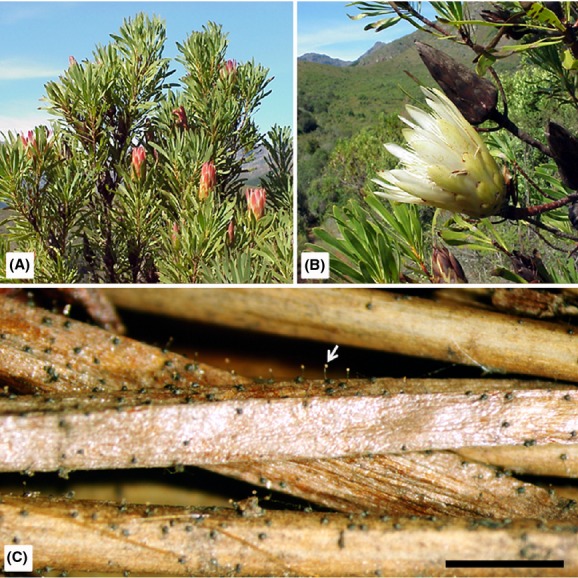
Overview of *Protea repens* and its fungal associate, *Knoxdaviesia proteae*. (A) *Protea repens* tree with light pink inflorescences in bloom, (B) cream inflorescence and infructescence (seed head) of *P. repens*, (C) *Knoxdaviesia proteae* sexual structures with visible spore droplets (arrow) on *P. repens* flowers. Scale bar = 1 cm.

The manner in which *Knoxdaviesia* species are moved between *Protea* infructescences is poorly understood. In the *Protea*–ophiostomatoid fungus symbiosis, mites appear to be the primary vectors of fungal spores and beetles are believed to act as secondary vectors (Roets et al. 2007, 2009a, 2011b). Roets et al. (2009a) found that the small mite vectors easily move vertically between infructescences on the same *Protea* plant in search of new and moist environments. These authors also found that the mites are phoretic on beetles associated with *Protea* species and proposed that lateral movement to infructescences of other plants is facilitated by beetles carrying the mites (Roets et al. 2009a). Two arthropod vectors are, therefore, involved in the dispersal of *Protea*-associated ophiostomatoid fungi, probably acting as short- and long-distance dispersal agents, respectively. Because of the sticky spore droplets produced by these fungi (Fig.1) and their enclosed niche, dispersal via abiotic agents, such as air and water, is unlikely to occur.

*Protea* infructescences are formed after every flowering season when serotinous *Protea* species close their involucral bracts around the inflorescences (Fig.1). These brown, cone-shaped structures house the seeds and are maintained on the plants until severe stress or death triggers seed release (Rebelo 1995). During their lifetime, infructescences may be colonized by numerous arthropods and microorganisms, including ophiostomatoid fungi (Coetzee and Giliomee 1985; Roets et al. 2005, 2006; Marincowitz et al. 2008; Theron et al. 2012). New *Protea* infructescences that form after flowering are presumably colonized by ophiostomatoid fungi from older infructescences (Roets et al. 2009a). The fungal population in these infructescences should, therefore, represent a subset of the established populations in the older fruiting structures (Roets et al. 2006). If dispersal between infructescences within *Protea* trees was more frequent than between different trees, individual *Protea* trees would be expected to harbor genetically discrete groups of ophiostomatoid fungi. In contrast, if medium- to long-distance dispersal played an important role in the biology of these fungi, fungal migrants from infructescences on other trees would also colonize new infructescences.

The ecological role that the *Protea*-specific ophiostomatoid fungi play in the biology of these plants is unknown, but they are not known to have a harmful effect on *Protea* seeds. In an infructescence, ophiostomatoid fungi are consistently the most abundant colonizers (Lee et al. 2005), and it is therefore speculated that they outcompete and exclude other fungi (Roets et al. 2013). The poor performance of ophiostomatoid fungi in laboratory cultures also appears to be as a result of a specific attachment to the chemistry of their *Protea* hosts (Roets et al. 2011a). If a beneficial *Protea*–ophiostomatoid relationship were to exist, in which ophiostomatoid fungi prevent potentially pathogenic fungi from colonizing infructescences and the *Protea* provides a favorable environment, the survival of both the ophiostomatoid fungi and their *Protea* hosts would be directly linked. In this regard, understanding the relative importance of vertical and lateral dispersal as well as the overall dispersal capacity of the ophiostomatoid fungi is relevant. The distances over which spores are moved would determine the extent of gene flow, impacting on the diversity and adaptability of these fungi.

Population diversity and structure are also largely affected by the sexual reproductive strategy of fungi (homo- or heterothallic). Homothallic fungi are able to self-fertilize, whereas heterothallic fungi are self-sterile and require outcrossing with a strain of opposite mating type for reproduction. Homothallic fungi that self-fertilize infrequently would have a similar population structure to heterothallic fungi, one with high genotypic diversity and random allele association (Milgroom 1996). However, when haploid organisms, such as the ophiostomatoid fungi, undergo self-fertilization, the products of meiosis are genetically identical and the population would have a clonal structure (Fincham and Day 1963; Milgroom 1996; Moore and Novak Frazer 2002). Despite extensive research into *Protea*-associated ophiostomatoid fungi, the sexual reproductive aspect of their biology has not been clarified. Investigation of *Protea*-associated ophiostomatoid dispersal, diversity, and reproduction is, therefore, important to understand the role of these fungi in the unique ecosystem in which they are found.

Elucidation of how genetic diversity in *K. proteae* is structured within and/or across populations may reveal the processes responsible for shaping its evolution. These may include factors such as reproductive strategy, dispersal, and ecology (Epperson 1993; Chung et al. 2004). The primary aim of this study was to determine gene flow among *K. proteae* individuals in different *Protea repens* trees and different infructescences age classes, thus evaluating the extent of lateral and/or vertical migration of *K. proteae* across a *Protea* population. A second aim was to compare the genetic diversity between *K. proteae* individuals in differently aged infructescences to better understand the origin of fungi in newly formed infructescences.

## Materials and Methods

### Sampling

Sampling of *P. repens* infructescences was conducted in the Gouritz area, Western Cape Province, South Africa (−34.2062; 21.681217), during September and November 2012. The isolated stand of *P. repens* trees chosen for study was situated in an area of approximately three square kilometers. It was bordered by roads to the north and west, across from which no other *P. repens* trees occurred. Farmland, devoid of *P. repens*, bordered this stand to the east, and an irregular distribution of *P. repens* trees was situated to the south. Approximately 20 infructescences from the current (2012) and 20 from the previous (2011) flowering season were collected from each of 11 randomly chosen *P. repens* trees (therefore 440 infructescences in total). To prevent repeated isolation of the same individual, only one fungal isolate was maintained per infructescence. Fungal isolations, DNA extraction, and species identity verification followed methods described previously (Aylward et al. 2014).

### Microsatellite amplification

For each *K. proteae* isolate, 12 microsatellite markers (Aylward et al. 2014) were amplified in three multiplex reactions with the KAPA2G Fast Multiplex PCR Kit (Kapa Biosystems, Inc., Boston, MA). The 25 *μ*L reactions contained 12.5 *μ*L KAPA2G, 1 mmol/L additional MgCl_2_, 20 ng DNA, and a variable concentration of primers (Table S1). PCR conditions were 3 min at 95°C followed by 27 cycles of: 15 sec at 95°C, 30 sec at 60°C, and 1 min at 72°C. The final extension was 30 min at 72°C. Each PCR plate contained a negative and positive control to indicate contamination and to standardize genotyping, respectively. The amplified products were subjected to a post-PCR clean-up and resolved on a 96-capillary Applied Biosystems 3730xl DNA Analyzer using a GeneScan 500 LIZ size standard (Applied Biosystems, Carlsbad, CA). Allele calling was done with GeneMarker 2.4.0 (Softgenetics LLC, State College, PA).

### Genetic diversity

The descriptive diversity indices for fungal isolates occurring in individual *P. repens* trees and the fungal population as a whole were computed using GenAlEx 6.501 (Peakall and Smouse 2006, 2012). Nei's (1978) unbiased estimate of expected heterozygosity (*H*_E_) was calculated using the frequency (*p*) of each allele (*i*) and the sample size (*n*) using the formula 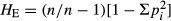. This index gives the probability that two randomly sampled individuals will be different (Nei 1978; NRC 1996). Heterozygosity is typically used to reflect genetic diversity and infer measures of differentiation, but its nonlinearity causes inaccuracies when polymorphism is high (Jost 2008). Therefore, a linear metric (Jost 2008) was also employed to describe diversity and calculate differentiation (see below).

To measure genetic diversity, the number of effective alleles (*N*_*e*_) was calculated using *N*_*e*_ = 1/1 - *h* (Kimura and Crow 1964; Brown and Weir 1983), where *h* is the expected heterozygosity 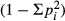 (Nei 1973). Stoddart and Taylor's (1988) genotypic diversity (G) was determined according to the formula *G* = 1/ ∑ [*f*_*x*_(*x*/*n*)^2^], where *f*_*x*_ is the number of distinct microsatellite multilocus genotypes occurring *x* times and *n* is the sample size. This index was used to obtain the maximum percentage of genotypic diversity (Ĝ) with the formula 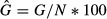 (McDonald et al. 1994), where *N* is the population size. The distribution of genotypes was investigated by calculating the evenness index (E5) as applied by Grünwald et al. (2003), using poppr, a package implemented in R 3.0.2 (R Development Core Team 2008; Kamvar et al. 2013).

### Relatedness among individuals

The relatedness of *K. proteae* individuals was investigated with the molecular variance parsimony technique by calculating pairwise distances between the microsatellite genotypes in Arlequin 3.5.1.3 (Excoffier and Lischer 2010). These distances were used to construct a minimum spanning network (MSN), containing all possible connections, in HapStar 0.7 (Teacher and Griffiths 2011). The hypothesis of random recombination was tested by investigating multilocus linkage disequilibrium in Multilocus 1.3b (Agapow and Burt 2001). A modified version of the index of association (*I*_*A*_), 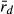 (Brown et al. 1980), was calculated and compared to a distribution of 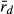 for 1000 simulated random datasets. A previous study showed that these 12 microsatellite loci are not in linkage disequilibrium (Aylward et al. 2014).

### Population differentiation

Diversity ratios to describe population differentiation were computed using smogd 1.2.5 (Crawford 2010). However, as smogd assumes a diploid organism and *K. proteae* is a haploid fungus, the estimated parameters that incorporate sample size and ploidy were calculated independently by substituting 2N for 1N in the Nei and Chesser (1983) formulas. The diversity present between subpopulations (Δ_ST_) represents the effective number of subpopulations and is the ratio of true diversity (Δ_T_; effective number of alleles in the total population) to the within-subpopulation diversity (Δ_S_). The inverse (Δ_S_/Δ_T_) of this ratio describes the proportion of diversity that is contained within the average subpopulation. It is a measure of similarity that will decrease as differentiation increases (Jost 2008).

The haploid estimate (*D*_est(hap)_) of relative differentiation (Jost's D) was calculated using *D*_est(hap)_ = [(*H*_*T_*est(hap)_ − (*H*_*S_*est(hap)_)/1 - *H*_*S_*est(hap)_)][*n*/(*n* − 1)] (Jost 2008). *H*_T_est(hap)_ and *H*_S_est(hap)_ are Nei and Chesser's (1983) estimates of total and mean expected subpopulation heterozygosity, respectively, adjusted for haploids and *n* is the number of subpopulations. An estimate analogous to *F*_ST_, the conventional measure of population differentiation (Weir and Cockerham 1984), was calculated with Multilocus 1.3b (Agapow and Burt 2001) and is given by *θ* = *Q* − *q*/1 − *q*, where *Q* is the probability that two alleles within a population are identical and *q* is the probability that two alleles from different populations are identical. Gene flow (*N*_*m*_) was estimated in PopGene 1.32 (Yeh et al. 1999) from *G*_ST_: *N*_*m*_ = 0.5(1−*G*_ST_)/*G*_ST_ (Slatkin and Barton 1989), where *G*_ST_ is a measure of differentiation relative to the total population (Nei 1973).

Analysis of molecular variance (AMOVA) was conducted with Arlequin 3.5.1.3 (Excoffier and Lischer 2010). This test is based on the premise that total molecular variance can be divided into different covariance components within a hierarchical context (within populations, among populations, and among groups of populations) (Excoffier et al. 1992). For this purpose, an F_ST_-like distance matrix and 10,000 permutations to test significance were used.

### Population structure

Structure 2.3.4 (Pritchard et al. 2000; Falush et al. 2003; Hubisz et al. 2009) was used to determine the number of clusters (*K*) in the population and to assign individuals to these clusters. Structure implements a Bayesian, model-based approach to cluster individuals based on their allelic frequencies when *K* is known. Twenty independent runs were conducted for *K* values between one and 10, using 500,000 burn-in and 750,000 Markov chain Monte Carlo repetitions, assuming an admixture model with correlated allele frequencies. Runs were initially conducted without supplying information about the population of origin, after which this information was included with the locprior model. The online platform Structure Harvester (http://taylor0.biology.ucla.edu/structureHarvester/) (Earl and von Holdt 2012) was used to compute L(*K*) (the mean log-likelihood of *K*) and Δ*K* (Evanno et al. 2005) to determine the optimal number of clusters.

## Results

### Genetic diversity

A total of 92 *K. proteae* isolates were obtained from the sampled *P. repens* infructescences. The low number of fungal individuals obtained relative to the high number of infructescences sampled is explained by arthropod damage to infructescences and the fact that *Knoxdaviesia* species are difficult to isolate. Although *Knoxdaviesia* species flourish within their natural environment, they grow slowly in culture and are easily overgrown by fungal contaminants that are present in the infructescences. Inconsistent sample sizes were therefore obtained for the different trees and different aged infructescences (Table S2), even after a second round of sampling in an attempt to increase numbers. In 10 of the loci, the proportions of null alleles (alleles for which no amplification product was directly observed) were low (between zero and 4.3%) and were treated as missing data in subsequent analyses. Loci KX6 and KX9 displayed high null allele percentages (36% and 46%, respectively) and were excluded from analyses. Their exclusion, however, did not significantly impact other diversity indices (Table1). Also, a plot of the number of sampled loci against the number of genotypes, calculated with Multilocus (Agapow and Burt 2001), began to plateau at 9 loci, indicating that the number of loci used was sufficient to capture the diversity of the population (data not shown).

**Table 1:** Number of alleles and diversity indices for all 12 loci.

Locus	*N*_a_^1^	Null alleles (%)	*N*_*e*_^2^	*H*_E_^3^
KX1	23	0	14.75	0.942
KX2	14	4.3	5.05	0.811
KX3	11	0	2.83	0.654
KX4	17	0	3.55	0.726
KX5	8	4.3	5.32	0.821
KX6	12	35.9	6.45	0.859
KX7	4	0	2.08	0.525
KX8	8	0	2.49	0.605
KX9	13	45.7	5.08	0.820
KX10	12	1.1	4.96	0.807
KX11	11	1.1	3.92	0.753
KX12	10	1.1	4.74	0.798
Mean ± SEM	11.92±1.38	8.00±4.51	5.10±0.95	0.76±0.03
Excluding KX6 & KX9	11.80±1.57	1.2±0.55	4.97±1.14	0.74±0.04

1 *N*_a_ = number of alleles.

2 *N*_*e*_ = Kimura and Crow's (1964) number of effective alleles; *N*_*e*_ = 1/1 − *h*.

3 *H*_*e*_ = Nei's unbiased expected heterozygosity; 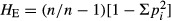.

**Figure 2:**
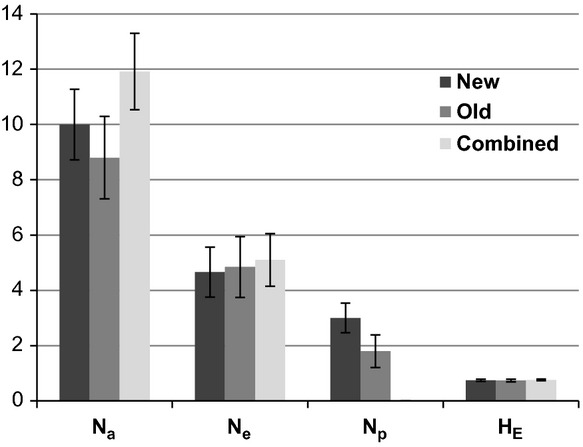
Comparison between the mean genetic diversity indices of *Knoxdaviesia proteae* individuals in new and old infructescences across 10 microsatellite loci. *N*_*a*_ = the total number of alleles, *N*_*e*_ = the number of effective alleles, *N*_*p*_ = the number of private alleles and *H*_E_ = Nei's (1978) unbiased estimate of expected heterozygosity. Error bars represent the standard errors of the mean. A *t*-test for independent samples showed no significant differences between the indices calculated for the different groups (new, old and combined). This is specifically relevant for *N*_*a*_ and *N*_*e*_, because it indicates that the genetic composition of all three groups is similar.

Across the 10 loci, a total of 118 alleles were detected with an average of 11.80 ± 1.57 alleles per locus. Allele frequencies ranged from 0.011 to 0.598, and the expected heterozygosity of the entire population across the 10 loci was 0.74 ± 0.04 (Table1). The genetic diversity or number of effective alleles (*N*_*e*_) was 4.97 ± 1.14. A *t*-test for independent samples implemented in Statistica 11 (StatSoft Inc 2012) did not reveal a significant difference between the diversity measures of isolates from new and old infructescences (Fig.2). The genetic diversities and genetic composition of the two groups were therefore similar. Among the 92 *K. proteae* isolates, 91 different genotypes were observed – the two identical genotypes originating from two different old infructescences on the same tree. This yielded a high maximum percentage of genotypic diversity (97.9%; G = 90.04) and a nearly maximum evenness value (E_5_) of 0.994.

### Relatedness among individuals

**Figure 3:**
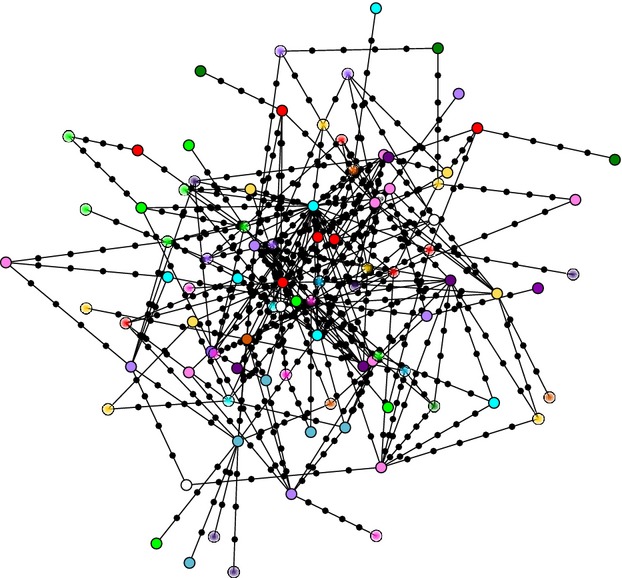
A minimum spanning network based on the most parsimonious pairwise distances between the 91 unique genotypes (nodes) in the *Knoxdaviesia proteae* population. Black circles represent missing genotypes between samples. Colors specify sampling locations – each color represents a different *P. repens* plant. Solid fills indicate isolates from new infructescences; gradient fills indicate isolates from old infructescences. The large amount of loops in the network suggests the presence of recombination and homoplasies in the population.

The MSN did not form apparent clusters (Fig.3), and loops were prevalent. Many genotypes therefore had more than one possible ancestor, suggesting the existence of numerous homoplasies and recombination (Posada and Crandall 2001). This was also evident from the results of the multilocus linkage disequilibrium analysis. The 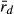 value of *K. proteae* lies within the range of the normal distribution and is not significantly different (*P* > 0.05) from zero (Fig. S1), which is indicative of a randomly recombining population. The numerous connections between the different haplotypes in the MSN and the lack of linkage disequilibrium between loci (Aylward et al. 2014) both imply that recombination between individuals is not restricted.

### Population differentiation

For calculations of population differentiation (Table2), two different scenarios were considered: (1) Individuals from new and old infructescences, respectively, group together (2 subpopulations) and (2) individuals from different *P. repens* trees group together (11 subpopulations). The differentiation indices calculated for both scenarios describe a situation in which the subpopulations account for all the genetic diversity. Δ_ST_ shows that there is almost no difference between the number of effective alleles (*N*_*e*_) in the total population and *N*_*e*_ in the subpopulations. The number of effective subpopulations therefore slightly exceeds one, and Δ_S_/Δ_T_ indicates that 99% (scenario 1) and 89% (scenario 2) of the diversity is already present in the respective subpopulations. Therefore, combining all subpopulations did not greatly increase the observed diversity. This was supported by the low values of *D* and the null value of the sample size-corrected estimate of *D*, *D*_est(hap)_, indicating that no differentiation exists between the subpopulations for each scenario. The nonsignificant values of theta for scenarios 1 and 2 were also congruent with the results of *D* and *D*_est(hap)_. Overall, these indices showed that there is no structuring of *K. proteae* individuals in this *P. repens* stand, but that all individuals belong to the same subpopulation. The lack of population differentiation (*D*_est(hap)_ = 0 and *θ* = 0) can be explained by the number of migrants (*N*_*m*_) in each generation. This measure was very high (*N*_*m*_ = 47.83) between old and new infructescences. Although much lower (*N*_*m*_ = 2.96), the value of *N*_*m*_ between *P. repens* trees remains greater than one, which is sufficient to prevent differentiation (McDermott and McDonald 1993).

**Table 2:** Descriptive measures of population differentiation for the two different subpopulation scenarios. Mean values and the standard error of the mean across the 10 loci are reported.^1^

	Scenario 1	Scenario 2
Ñ^2^	44.93	6.69
*N*_*e*_^3^	4.75±0.02	3.22±0.14
Δ_ST_^4^	1.01±0.00	1.13±0.03
Δ_S_/Δ_T_^5^	0.99±0.00	0.89±0.02
*D*^6^	0.02±0.01	0.13±0.02
*D*_est (hap)_^7^	0	0
*θ*^8^	0	0.01
*G*_ST_^9^	0.01	0.14
*N*_*m*_^10^	47.83	2.96

1 Scenario (1) individuals from new versus old infructescences; (2) individuals from different *P. repens* trees.

2 Ñ = harmonic mean of the sample sizes.

3 *N*_*e*_ = Kimura and Crow's (1964) number of effective alleles; *N*_*e*_ = 1/1 – *h*.

4 Δ_ST_ = diversity between subpopulations or the effective number of subpopulations.

5 Δ_S_/Δ_T_ = proportion of diversity in a subpopulation.

6 *D* = actual (relative) differentiation.

7 *D*_est(hap)_ = the haploid estimate of *D*; *D*_est(hap)_ = [(*H*_*T*_est(hap)_ − *H*_*S*_est(hap)_)/(1 − *H*_*S*_est(hap)_)][*n*/(*n* − 1)].

8 *θ* = conventional measure of relative differentiation; *θ* = *Q* − *q*/1 – *q*.

9 *G*_ST_ = gene differentiation relative to the total population (Nei 1973).

10 *N*_*m*_ = estimated gene flow; *N*_*m*_ = 0.5(1 − *G*_ST_)/*G*_ST._

For the AMOVA (Table3), *K. proteae* individuals isolated from each age class of infructescence on every different *P. repens* tree (Table S2) were considered as one population (therefore 22 subpopulations) and populations were grouped according to the two possible scenarios mentioned above. The AMOVA results supported those obtained using the indices of Jost (2008), showing that more than 96% of molecular variation is contained within the subpopulations. Very little, but significant (*P* < 0.01), differentiation was detected among the subpopulations relative to the total population (*θ*_ST_) and among subpopulations grouped based on age class or trees sampled (*θ*_SC_). Differentiation was, however, no longer significant (*P* > 0.7) at the highest hierarchy when infructescence age classes or trees were compared (*θ*_CT_). This showed that the basic subunits (subpopulations) of the *K. proteae* population are highly diverse and, together, form a genetically cohesive population. As no differentiation was observed between infructescence age classes or trees, these groups must be connected by gene flow as has been indicated by *N*_*m*_ (Table2).

**Table 3:** Analysis of molecular variance (AMOVA) results showing the variance attributable to each hierarchy^1^ in the Gouritz *Knoxdaviesia proteae* population.

Variance component	df	Variance	% total	*P*^2^	Fixation
*Scenario 1*					
Among infructescence age classes	1	0	0	0.786	*θ*_CT_ = 0
Among subpopulations within infructescence age classes	19	0.135	3.77	<0.01	*θ*_SC_ = 0.035
Within subpopulations	71^3^	3.480	96.74	<0.01	*θ*_ST_ = 0.033
*Scenario 2*					
Among *P. repens* trees	10	0	0	0.822	*θ*_CT_ = 0
Among subpopulations within trees	10	0.176	4.88	<0.01	*θ*_SC_ = 0.048
Within subpopulations	71^3^	3.480	96.58	<0.01	*θ*_ST_ = 0.034

1 The hierarchical structure of this population is built on 22 subpopulations comprised of all *K. proteae* individuals isolated from a specific age class in a specific *P. repens* tree. The two scenarios group these subpopulations in different ways for subsequent analyses. Scenario 1 first compares them within their infructescence age classes (new and old) and then among the age classes (new vs. old). Scenario 2 compares the two subpopulations present within each tree to each other and then compares the 11 different trees.

2 The probability of obtaining a more extreme variance and fixation index by chance.

3 As no *K. proteae* isolates could be obtained from the old infructescence of tree 3, degrees of freedom are 71 instead of 72.

### Population structure

The same scenarios described above were implemented when running Structure with the locprior model. Two different locprior runs were therefore conducted, each assuming different populations of origin. The Δ*K* values of the runs both without and including sampling information highlighted *K* values between four and nine as the most likely. However, it is important to consider that Δ*K* cannot evaluate *K* = 1 and the maximum log-likelihood of *K*, L(*K*), was always observed at *K* = 1. Evanno et al. (2005) also noted that the variance in the mean L(*K*) begins to increase after the correct *K* value is reached, which was observed in the data for *K* ≥ 2. Inspection of the clusters highlighted by Δ*K* revealed that they do not provide information on population structure, but rather distribute similar proportions of the individuals' genetic material to all of the clusters. The formation of such apparently uninformative clusters and Δ*K* values are similar to observations by Waples and Gaggiotti (2006) for a simulated dataset with high gene flow. Mean alpha values for the runs were always greater than one, signifying high levels of admixture between individuals (Falush et al. 2003). A value of *K* = 1 is therefore the most likely and biologically meaningful when considered together with the population differentiation statistics.

## Discussion

While plants and animals in the CCR are well known and have been intensively studied, there is a relatively sparse knowledge of the microbes in this unique and iconic ecosystem (Lee et al. 2004; Crous et al. 2006; Marincowitz et al. 2008; Slabbert et al. 2010). Ironically, while there is concern that the microbial biodiversity of this and other important ecosystems tends to be overlooked, there is even less knowledge relating to the biology of these microbes (Cowan et al. 2013). Although the biology and genetics of non-*Protea* ophiostomatoid fungi have been extensively studied, this study represents the first attempt to understand the interspecific genetic diversity of any fungus in the CCR. The results have shown intriguing patterns that advance our understanding of an interesting fungus in the ophiostomatoid assemblage, not only in this ecosystem, but also relating to these fungi globally.

The high level of genetic diversity found for *K. proteae*, a native fungus in the CCR, is not surprising. The genotypic diversity of *K. proteae*, however, far exceeds that reported in previous studies of ophiostomatoid fungi with similar dual vector systems (Barnes 2002; Zhou et al. 2007; Nkuekam et al. 2009). This high level of diversity in *K. proteae* appears to be the result of regular gene flow and outcrossing. Importantly, the similarity in genetic composition and the exceptionally high gene flow between *K. proteae* individuals from old and new infructescences supports the findings of Roets et al. (2006, 2009a) that new infections by *K. proteae* found in fresh infructescences originate from the infructescences of previous years that remain on the trees.

Although this study presents support for fungal migration from old to new infructescences (vertical transmission) and *K. proteae* individuals in new infructescences are therefore the offspring of those in old infructescences, this parent–offspring relationship is not restricted to individual trees. This is evident from the lack of genetic cohesion within individual *P. repens* trees, which suggests that vertical migration is not the primary method by which gene flow is achieved. Instead, the observed population structure rather emphasizes medium- to long-distance dispersal of *K. proteae*. Thus, a *K. proteae* individual in a given infructescence may have the potential to disperse to any other infructescence in the *P. repens* population.

The results of this study revealed fungal panmixia within a *P. repens* stand. Panmixia has previously been reported for several pathogenic (Zeller et al. 2003; Pringle et al. 2005; Groenewald et al. 2008; Rypien et al. 2008) and one endophytic ascomycete fungus (Wickert et al. 2012); however, population studies of fungi in the ophiostomatoid assemblage often show structured populations (Morin et al. 2004; Lee et al. 2007; Tsui et al. 2012). The panmictic population structure of this fungus suggests that frequent random dispersal between *P. repens* trees and a recombining reproductive strategy predominates within *K. proteae*. Due to the high mobility of *K. proteae* and the frequent dispersal events, a *K. proteae* population cannot be defined as occurring within a single *P. repens* tree or infructescence age class, but rather as occupying a stand of *P. repens* trees.

At least two avenues are available for short- and long-distance dispersal of ophiostomatoid fungi – mites and beetles. Mites are known to leave previous-year infructescences and self-disperse upwards to new, moist infructescences formed the following year (Roets et al. 2009a). However, as the primary vectors of ophiostomatoid fungi, the predisposition of mites to phoresy greatly affects the fungal population structure. These small arthropods have been found to be phoretic on numerous other organisms, including other arthropods, insects, and birds (Proctor and Owens 2000; Krantz and Walter 2009). The large numbers of ophiostomatoid-fungus mite vectors that have been found on beetles (Roets et al. 2009a) also show an inclination of mites to utilize larger organisms to facilitate long-distance dispersal. The apparently panmictic structure of *K. proteae* in a stand of *P. repens* trees highlights the importance of long-distance dispersal and therefore the role of beetles as ophiostomatoid vectors and mite vehicles. The mite-vectoring beetles have also been implicated as *Protea* pollinators (Coetzee and Giliomee 1985), and as such, they visit numerous inflorescences carrying fungus-vectoring mites as well as pollen from plant to plant. This activity may explain the high levels of gene flow observed between different *P. repens* trees.

Associations between microorganisms and arthropods are widespread and well known, especially in pathogenic systems such as malaria (Sinden 2002) and Lyme disease (Derdakova and Lencakova 2005). Symbiotic relationships between fungi, mites, and insects such as beetles, ants, and bees are also well known and have recently been reviewed (Hofstetter and Moser 2014). Compared with these systems, the multivectored dispersal of *Protea*-associated ophiostomatoid fungi, however, seems exceptional as mites and beetles do not provide two isolated mechanisms of dispersal, but rather apply a hierarchical method to achieve short- and long-distance dispersal (Roets et al. 2009a). Many of the Northern Hemisphere beetle–ophiostomatoid associations also include mites, forming a multivector system (Moser and Roton 1971; Moser 1985; Moser et al. 2010), although not necessarily a hierarchical one. The reproductive strategies of fungi in these multivectored systems vary; outcrossing is often encountered and even some homothallic species have recombining populations (Zhou et al. 2007; Marin et al. 2009), whereas other species, such as *Ophiostoma novo-ulmi* Brasier and *O. quercus* (Georgévitch) Nannf., prefer self-fertilization or asexual reproduction (Brasier 1988; Grobbelaar et al. 2009). Outcrossing is, however, not necessarily associated with high levels of diversity (Zhou et al. 2007; Tsui et al. 2012). *Ophiostoma piceae* (Münch) Syd. & P. Syd. appears to be the only ophiostomatoid fungus investigated to date where the population genetics reflects that of *K. proteae*, showing little differentiation, outcrossing, and very high genetic diversity (Gagné et al. 2001).

## Conclusions

This study has shown that *K. proteae* in the CCR is characterized by exceptional genetic and genotypic diversity. The diversity appears to be maintained by high levels of gene flow that prevents population differentiation, thus limiting the effects of genetic drift. This suggests that a panmictic population of *K. proteae* exists within *P. repens* stands in close proximity to each other. Consequently, the role of beetles in the dispersal of *Protea*-associated ophiostomatoid fungi appears to be essential, because they facilitate transport between *Protea* trees and would, therefore, be primarily responsible for the observed panmixia.

Although the extent of vertical and lateral dispersal in *K. proteae* has been addressed in this study, the lack of population structure observed prompts further questions. The geographic range over which panmixia is maintained in *K. proteae* is specifically interesting and will likely be a function of the migration capacity of the long-distance beetle vectors. Furthermore, the ecological role of the ophiostomatoid fungi in this unusual and interesting niche has not yet been elucidated.

## Data Accessibility

Microsatellite genotypes, sampling information, STRUCTURE example input and paramater files and alpha values are available as online supporting information.

## Supporting Information

**Table S1.** Primer concentrations in the three multiplex reactions used to genotype *K. proteae*. **Figure. S1** Histogram depicting the distribution of  in *K. proteae* for 1 000 randomizations.

**Data S1.** Microsatellite genotypes of *K*. *proteae* individuals.

**Data S2.** Sampling locations and number of *K. proteae* isolates obtained from each *P. repens* tree.

**Data S3.** Example of a STRUCTURE input file.

**Data S4.** Example of a STRUCTURE main parameter file.

**Data S5.** Average alpha values inferred from clustering analyses in STRUCTURE.

**Data S6.** Explanation of the content of the data files.
