# Supplementary material for: Panmixia defines the genetic diversity of a unique arthropod-dispersed fungus specific to Protea flowers
Source: Ecol Evol. 2014 Aug 21;4(17):3444–55. doi: 10.1002/ece3.1149 (PMC4228618; doi:10.1002/ece3.1149)
Supplement: Supplementary file 1 [file ece30004-3444-sd1.docx]

Table S1 Primer concentrations in the three multiplex reactions used to genotype *K. proteae*

| Reaction 1 | | |  | Reaction 2 | | |  | Reaction 3 | | |
| --- | --- | --- | --- | --- | --- | --- | --- | --- | --- | --- |
| Locus | Primers | Concentration (nm) |  | Locus | Primers | Concentration (nm) |  | Locus | Primers | Concentration (nm) |
| KX1 | *KX1-1* | 20 |  | KX2 | *KX2-1* | 60 |  | KX5 | *KX5-1* | 40 |
|  | *KX1-2* | 20 |  |  | *KX2-2* | 40 |  |  | *KX5-2* | 40 |
| KX3 | *KX3-1* | 20 |  | KX4 | *KX4-1* | 20 |  | KX6 | *KX6-1* | 40 |
|  | *KX3-2* | 20 |  |  | *KX4-2* | 20 |  |  | *KX6-2* | 40 |
| KX10 | *KX10-1* | 20 |  | KX7 | *KX7-1* | 40 |  | KX8 | *KX8-1* | 40 |
|  | *KX10-2* | 40 |  |  | *KX7-2* | 20 |  |  | *KX8-2* | 40 |
| KX11 | *KX11-1* | 40 |  | KX12 | *KX12-1* | 40 |  | KX9 | *KX9-1* | 40 |
|  | *KX11-2* | 20 |  |  | *KX12-2* | 40 |  |  | *KX9-2* | 40 |

Fig. S1 Histogram depicting the distribution of $\bar{r}_{d}$ in *K. proteae* for 1 000 randomizations. The observed value of $\bar{r}_{d}$ and the P-value (shown on the graph) indicate that there is no significant difference from zero, supporting the hypothesis of random recombination.
